# Factors associated with vaccination against Covid-19 in pregnant and hospitalized postpartum women: A retrospective cohort study

**DOI:** 10.1371/journal.pone.0269091

**Published:** 2022-06-15

**Authors:** Marcela de Andrade Pereira Silva, Helena Fiats Ribeiro, Rosana Rosseto de Oliveira, Fernando Castilho Pelloso, Constanza Pujals, Raíssa Bocchi Pedroso, Maria Dalva de Barros Carvalho, Sandra Marisa Pelloso

**Affiliations:** 1 Department of Post Graduate in Health Science, State University of Maringa, Maringa, Parana, Brazil; 2 Departament of Nursing, State University of Maringa, Maringa, Parana, Brazil; 3 Departament of Medicine, Federal University of Parana, Curitiba, Parana, Brazil; University of Palermo: Universita degli Studi di Palermo, ITALY

## Abstract

**Objective:**

To analyze the characteristics associated with vaccination against Covid-19 in pregnant and postpartum women with Severe Acute Respiratory Syndrome in Brazil and to investigate a possible association between vaccination and the clinical course and outcome of the disease.

**Methods:**

Retrospective cohort study of hospitalized pregnant and postpartum women diagnosed with Severe Acute Respiratory Syndrome (SARS) by SARS-CoV-2, presenting onset of signs and symptoms between May and October 2021. Secondary data were used, available in the Influenza Epidemiological Surveillance Information System (SIVEP-Gripe). Data were analyzed using the SPSS statistical program, medians were applied to present continuous variables and frequencies, and proportions were calculated for categorical variables, using logistic and multivariate regression analysis.

**Results:**

The final study population included 3,585 pregnant and postpartum women, of whom 596 (16.6) were vaccinated: 443 (74.3%) received one dose and 153 (25.7%) received two doses. They were factors associated with non-vaccination against Covid-19 age ≤ 19 anos (OR: 2.57; IC_95%_ 1.40;4.71), non-white women (OR: 1.34; IC_95%_ 1.07;1.67) and those who required ventilatory support (OR: 1.51; IC_95%_ 1.19;1.90) and invasive ventilation (OR: 2.05; IC_95%_ 1.37;3.08). On the other hand, vaccination was associated with advanced maternal age (OR: 0.60; IC_95%_ 0.48;0.76), presence of comorbidities (OR: 0.57; IC_95%_ 0.45;0.72) and loss of taste (OR: 0.63; IC_95%_ 0.48;0.82).

**Conclusions:**

Demographic, ethnic-racial and clinical characteristics were associated with the vaccination status of pregnant and postpartum women with SARS by SARS-CoV-2 in Brazil. Vaccination against Covid-19 in the obstetric population has already shown positive results in the evolution of severe cases, which reiterates its importance. It is essential that health services advance vaccination against Covid-19 in the obstetric population, especially adolescentes and non-white women.

## Introduction

In December 2019, a new coronavirus (SARS-CoV-2) was identified in the city of Wuhan in China, the causing agent of Covid-19, an acute respiratory disease with a clinical spectrum that ranges from asymptomatic to severe infections. Given its high transmissibility and rapid geographic spread, on March 30, 2020, the World Health Organization (WHO) declared a pandemic status, which resulted in a major challenge for public health authorities worldwide [1]. After 24 months of the pandemic, there were already 485 million cases and 6.1 million deaths worldwide [2].

It is known that the obstetric population is an at-risk group, with a high rate of admission to the intensive care unit (ICU), need for supplemental oxygen and mortality [3, 4]. Brazil has been the country with the highest number of maternal deaths due to Covid-19 worldwide, with a case fatality rate of 7.2% [5]. Faced with this catastrophic pandemic, the scientific community began to develop vacines against Covid-19 [6, 7].

Moreover, pregnant women were excluded from phase 3 clinical trials of Covid-19 vaccines, resulting in limited data on their efficacy and safety during pregnancy and postpartum. Because of this, in January 2021, the WHO recommended vaccination only for pregnant women who had an unavoidable high risk of exposure, such as health professionals or pregnant women with comorbidities due to the increased risk of serious illness [8].

Nevertheless, in light of new studies [8, 9], and considering that the risks of Covid-19 in the obstetric population outweigh the undocumented and hypothetical risks of Covid-19 vaccines in pregnancy, medical organizations and committees including the American College of Gynecology and Obstetrics (ACOG) [10], Center for Disease Control and Prevention (CDC) [11], the International Federation of Gynecology and Obstetrics (FIGO) [12], and the Brazilian Ministry of Health [13] recommend its use among pregnant and postpartum women.

Given this context and the importance of understanding the impact of vaccination against Covid-19 in the obstetric population, this article aims to analyze the characteristics associated with vaccination against Covid-19 in pregnant and postpartum women with Severe Acute Respiratory Syndrome in Brazil and investigate possible association between vaccination and the clinical course and outcome of the disease.

## Methods

A retrospective cohort study of hospitalized pregnant and postpartum women diagnosed with Severe Acute Respiratory Syndrome (SARS) by SARS-CoV-2. The Ministry of Health defines SARS as the individual with flu-like syndrome who has dyspnea/respiratory discomfort, persistent pressure in the chest or O2 saturation lower than 95% in ambient air or cyanosis [14].

Secondary, non-nominal data available in the Influenza Epidemiological Surveillance Information System (SIVEP-Gripe) were used. SIVEP-Gripe was developed in 2009 by the Ministry of Health for SARS surveillance in Brazil, and in 2020 it was redesigned, incorporating the surveillance of SARS cases by SARS-CoV2, currently being the official system for recording SARS cases and deaths in the country. The database was downloaded on November 5, 2021 using the R software.

In Brazil, the National Vaccination Campaign against Covid-19 started on January 18, 2021, however, pregnant and postpartum women were included as a priority group only in the 6th edition of the National Plan for the Operationalization of Vaccination against Covid-19 in Brasil [13], published on April 28, 2021. The study population consisted of pregnant and postpartum women who had the onset of signs and symptoms between May and October 2021 (Epidemiological week 18 to 43).

The cases analyzed comprised the 27 federative units of Brazil, with an estimated population of 213.3 million in 2021. The age group of 10 to 49 years old was delimited for the study population, given in Brazil it is considered a woman in fertile age the one belonging to this group [15]. Cases in which there was no information about the patient’s vaccination status in relation to vaccination against Covid-19, as well as cases in which there was no date for the application of the immunobiological were excluded.

Demographic, ethnic-racial, obstetric characteristics and comorbidities, as well as clinical characteristics and evolution were compared between women who received at least one dose of Covid-19 vaccine before the onset of signs and symptoms, regardless of the type of immunobiological received, and women unvaccinated.

The following independent variables were analyzed: maternal age (≤ 19 anos; 20 a 34 anos; ≥ 35 anos), obstetric population (pregnant; postpartum women), gestational trimester, race/ethnicity (white; non-white), presence of comorbidities such as: diabetes mellitus, chronic cardiovascular disease, chronic liver disease, chronic neurological disease, chronic kidney disease, chronic hematologic disease, immunodeficiency, asthma and other lung diseases, obesity and obstetric complications including gestational diabetes mellitus, gestational hypertension/pre-eclampsia/eclampsia, and gestational hypothyroidism.

The clinical characteristics analyzed were: symptoms of measured fever, cough, sore throat/odynophagia, dyspnea, respiratory distress, O2 saturation below 95%, diarrhea, vomiting, abdominal pain, fatigue, loss of smell (anosmia), loss of taste (ageusia) and others such as headache, myalgia, asthenia, coryza (nasal discharge), nasal congestion. Need for admission to the intensive care unit (ICU), length of stay in the ICU (≤ 7 days; > 7 days), need for ventilatory support, need for invasive ventilation, and outcome (cure; death).

Means and standard deviation were used to present continuous variables and frequencies and proportions for categorical variables were calculated. For logistic regression analysis, initially univariate analysis was performed using Pearson’s chi-square, considering p <0.05 as statistically significant. As a measure of association, the odds ratio (OR) with a confidence interval (CI) of 95% was used. For the multivariate analysis, the independent variables obtained by the univariate analysis with p <0.20 were considered, and the stepwise forward regression was chosen. The association measure used was the adjusted odds ratio (adjusted OR) with a confidence interval (CI) of 95%, respecting the absence of multicollinearity in the model. Data were analyzed using the SPSS statistical program (Statistical Package for the Social Sciences, version 23, IBM, USA).

All data were obtained from public databases (http://datasus.saude.gov.br/). Because it is public domain data, without the possibility of individual identification, this study does not need approval from na ethics committee, according to Brazilian standards [16].

## Results

5,598 SARS cases were reported in pregnant and postpartum women with onset of signs and symptoms between May and October 2021, of which 3,585 (64.0%) were considered eligible. 1,922 cases were excluded for not having information on the vaccination status against Covid-19 and 91 cases for not having a record of the immunobiological application date.

The final study population included 3,585 pregnant and postpartum women, of whom 596 (16.6%) were vaccinated: 443 (74.3%) received one dose and 153 (25.7%) received two doses. The application date of the first dose ranged from <1 to 38 weeks before the date of the first signs and symptoms, with a mean of 7 weeks (±7.4). Regarding the second dose, the application date ranged from <1 to 35 weeks before the date of the first signs and symptoms, with an average of 9 weeks (±8.3).

Demographic, ethnic-racial, obstetric characteristics and comorbidities according to vaccination status is shown in Table 1. Compared with unvaccinated women, vaccinated women were older, white and had comorbidities. The proportion of pregnant and postpartum women was equivalent, as well as the gestational trimester. In the univariate analysis, there was an association with vaccination status: age ≤ 19 years (OR: 2.99; IC_95%_ 1.76;5.10), age ≥ 35 years (OR: 0.61; IC_95%_ 0.51;0.73), race/ethnicity non-white (OR: 1.23; IC_95%_ 1.02;1.49), presence of comorbidities (OR: 0.64; IC_95%_ 0.53;0.78) (Table 1).

**Table 1:** Univariate analysis of demographic, ethnic-racial, obstetric characteristics and comorbidities of pregnant and postpartum women with SARS according to the vaccination status against Covid-19. Brazil, 2021.

Variables	n^a^ (%)	Vaccinated		Unvaccinated		Crude OR (IC95%)	p-value
		n	%	n	%		
Age (years)							
≤ 19	265 (7.4)	15	2.5	250	8.4	2.99 (1.76–5.10)	<0.001
20 a 34	2299 (64.1)	350	58.7	1949	65.2	1	
≥ 35	1021 (28.5)	231	38.8	790	26.4	0.61 (0.51–0.73)	<0.001
Race/ethnicity							
White	1594 (49.4)	283	53.8	1311	48.6	1	
Non-white	1632 (50.6)	243	46.2	1389	51.4	1.23 (1.02–1.49)	0.028
Obstetric Population							
Pregnant	2914 (81.3)	483	81.0	2431	81.3	1	
Puerpera	671 (18.7)	113	19.0	558	18.7	0.98 (0.78–1.22)	0.868
Gestational Age^b^							
1^st^ trimester	261 (9.3)	42	9.2	219	9.3	1	
2^nd^ trimester	813 (28.9)	134	29.2	679	28.8	0.97 (0.67–1.42)	0.882
3^rd^ trimester	1743 (61.9)	282	61.6	1459	61.9	0.98 (0.69–1.40)	0.934
Comorbities							
Yes	987 (27.5)	211	35.4	776	26.0	0.64 (0.53–0.78)	<0.001
No	2598 (72.5)	385	64.6	2213	74.0	1	

^a^Some information was ignored or blank, which justifies the “n” of some variables being different.

^b^Considering only the group of pregnant women.

The clinical evolution of pregnant and postpartum women according to vaccination status is shown in Table 2. Unvaccinated women tended to have symptoms of fever, cough, dyspnea, respiratory distress, O2 saturation below 95%, diarrhea and abdominal pain. Among vaccinated women, the presence of symptoms such as sore throat, vomiting, fatigue, loss of smell, loss of taste and others were more prevalent. The need for ICU hospitalization, ICU stay longer than 7 days, need for ventilatory support, need for invasive ventilation and evolution to death prevailed among unvaccinated women (Table 2).

**Table 2:** Univariate analysis of clinical characteristics and evolution of pregnant and postpartum women with SARS according to the vaccination status against Covid-19. Brazil, 2021.

Variables	n^a^ (%)	Vaccinated		Unvaccinated		Crude OR (IC95%)	p-value
		n	%	n	%		
Fever							
Yes	1851 (58.2)	289	55.7	1562	58.7	1.13 (0.94–1.37)	0.199
No	1328 (41.8)	230	44.3	1098	41.3	1	
Cough							
Yes	2619 (78.7)	429	78.0	2190	78.9	1.05 (0.84–1.31)	0.641
No	707 (21.3)	121	22.0	586	21.1	1	
Sore Throat							
Yes	828 (28.4)	143	29.4	685	28.2	0.94 (0.76–1.17)	0.593
No	2091 (71.6)	344	70.6	1747	71.8	1	
Dyspnea							
Yes	2205 (67.8)	317	59.1	1888	69.5	1.57 (1.30–1.90)	<0.001
No	1047 (32.2)	219	40.9	828	30.5	1	
Respiratory Distress							
Yes	1727 (55.6)	253	49.4	1474	56.8	1.35 (1.11–1.63)	0.002
No	1378 (44.4)	259	50.6	1119	43.2	1	
O2 Saturation <95%							
Yes	1649 (53.2)	221	43.5	1428	55.1	1.59 (1.31–1.93)	<0.001
No	1453 (46.8)	287	56.5	1166	44.9	1	
Diarrhea							
Yes	334 (11.8)	53	11.2	281	20.7	1.08 (0.79–1.47)	0.642
No	2500 (88.2)	422	88.8	1078	79.3	1	
Vomiting							
Yes	361 (12.7)	68	14.3	293	12.4	0.85 (0.64–1.12)	0.248
No	2475 (87.3)	406	85.7	2069	87.6	1	
Abdominal Pain							
Yes	294 (10.5)	43	9.2	251	10.7	1.18 (0.84–1.66)	0.328
No	2507 (89.5)	423	90.8	2084	89.3	1	
Fatigue							
Yes	1124 (37.9)	186	38.3	938	37.9	0.98 (0.80–1.20)	0.867
No	1839 (62.1)	300	61.7	1539	62.1	1	
Anosmia							
Yes	561 (19.6)	108	22.7	453	19.0	0.80 (0.63–1.02)	0.068
No	2294 (80.4)	368	77.3	1926	81.0	1	
Ageusia							
Yes	510 (17.9)	106	22.3	404	17.0	0.72 (0.56–0.91)	0.007
No	2336 (82.1)	370	77.7	1966	83.0	1	
Other symptoms							
Yes	1411 (48.8)	262	54.9	1149	47.6	0.75 (0.61–0.91)	0.004
No	1478 (51.2)	215	45.1	1263	52.4	1	
ICU admission							
Yes	1105 (32.5)	158	28.0	947	33.4	1.29 (1.06–1.58)	0.011
No	2292 (67.5)	407	72.0	1885	66.6	1	
ICU stay							
≤ 7 days	300 (44.0)	47	63.5	253	41.6	1	
> 7 days	382 (56.0)	27	36.5	355	58.4	2.44 (1.48–4.03)	<0.001
Ventilatory support							
Yes	1992 (60.1)	282	51.0	1710	62.0	1.57 (1.30–1.88)	<0.001
No	1320 (39.9)	271	49.0	1049	38.0	1	
Invasive Ventilation							
Yes	515 (15.5)	48	8.7	467	16.9	2.14 (1.57–2.93)	<0.001
No	2797 (84.5)	505	91.3	2292	83.1	1	
Outcome							
Cure	2705 (87.3)	450	91.6	2255	86.5	1	
Death	393 (12.7)	41	8.4	352	13.5	1.71 (1.22–2.40)	0.002

^a^Some information was ignored or blank, which justifies the “n” of some variables being different.

The univariate analysis showed an association with vaccination status: dyspnea (OR: 1.57; IC_95%_ 1.30;1.90), respiratory distress (OR: 1.35; IC_95%_ 1.11;1.63), O2 saturation lower than 95% (OR: 1.59; IC_95%_ 1.31;1.93), loss of taste (OR: 0.72; IC_95%_ 0.56;0.91), other symptoms (OR: 0.75; IC_95%_ 0.61;0.91), ICU admission (OR: 1.29; IC_95%_ 1.06;1.58), ICU stay longer than 7 days (OR: 2.44; IC_95%_ 1.48;4.03), need for ventilatory support (OR: 1.57; IC_95%_ 1.30;1.88), need for invasive ventilation (OR: 2.14; IC_95%_ 1.57;2.93) and death (OR: 1.71; IC_95%_ 1.22;2.40) (Table 2).

Women who required ventilatory support and invasive ventilation were 1.51 and 2.05 times more likely to be unvaccinated, respectively. The extremes of maternal age were associated with vaccination in different ways, where adolescents were 2.05 times more likely to be unvaccinated, while advanced maternal age proved to be a protective factor against non-vaccination. Loss of taste was the only symptom that remained independently associated with vaccination. As for race/ethnicity, it was identified that non-white women were 1.34 times more likely to be unvaccinated. Table 3 presents the multivariate logistic regression analysis.

**Table 3:** Multiple logistic regression analysis of factors associated with the vaccination status against Covid-19 in pregnant and postpartum women with SARS. Brasil, 2021.

Variables	n (%)	Adjusted OR (IC95%)	p-value
**Demographic characteristics and comorbidities**			
Age ≤ 19 years	265 (7.4)	2.57 (1.40–4.71)	0.002
Age ≥ 35 years	1021 (28.5)	0.60 (0.48–0.76)	<0.001
Non-white	1632 (50.6)	1.34 (1.07–1.67)	0.009
Comorbities	987 (27.5)	0.57 (0.45–0.72)	<0.001
**Clinical characteristics**			
Ventilatory support	1992 (60.1)	1.51 (1.19–1.90)	0.001
Invasive ventilation	515 (15.5)	2.05 (1.37–3.08)	<0.001
Loss of taste	510 (17.9)	0.63 (0.48–0.82)	0.001

## Discussion

The findings of this study show that maternal age ≤ 19 years, non-white women and who required ventilatory support and invasive ventilation were factors associated with non-vaccination against Covid-19 in pregnant and postpartum women with SARS by SARS-CoV-2 in Brazil. The factors associated with vaccination against Covid-19 were advanced maternal age, presence of comorbidities and the loss of taste symptom.

The Brazilian Ministry of Health, in its first edition of the National Vaccination Plan against Covid-19 published in December 2020 [17], contraindicated vaccination in pregnant women due to the lack of data on its efficacy and safety during pregnancy, however in its second edition, published in January 2021 [18], vaccination was recommended for pregnant women, as long as they belong to one of the priority groups and the decision-making is conditioned to a joint deliberation between the woman and the health team responsible for her care. Only in the sixth edition, published in April 2021, the population of pregnant and postpartum women were included as a priority group for vaccination [13].

However, vaccination against covid-19 in pregnant and postpartum women, without risk factors, was temporarily interrupted from 05/19 to 07/06/2021 in Brazil, after the notification of a post-vaccination thromboembolic adverse event in a pregnant woman. During this period, vaccination was maintained only for pregnant and postpartum women with comorbidities [19]. Thus, it is possible to understand the association identified in the present study between the presence of comorbidities and vaccination against Covid-19.

Studies have shown that pregnant and postpartum women with comorbidities are at high risk of developing more severe cases of Covid-19, in addition to unfavorable maternal-fetal outcomes [4, 20], which justifies the prioritization of vaccination for these women. However, in the current context, it is necessary to establish actions by public authorities and managers that guarantee the vaccination of the entire obstetric population, regardless of added risk factors, in addition to actions that increase this population’s adherence to vaccination, such as reducing barriers that make it difficult to access health services, spread adequate and evidence-based information by health professionals, and combat the circulation of false news.

With regard to maternal age, vaccination in adolescentes aged 12 to 17 years old started only in September 2021 in Brazil, after publication of the technique note n°45/2021 [21]. In view of this, the association identified in the present study in relation to maternal age ≤ 19 years and non-vaccination against Covid-19 is understandable, given that vaccination was not available for this public during a large extent of the study period. Regarding advanced maternal age (≥ 35 years) have been associated with vaccination, it is believed that is related to the presence of comorbidities, since the frequency of pre-existing chronic diseases increases with age, presenting higher proportions in women with advanced maternal age [22].

Studies published in the context of the Covid-19 pandemic show increased racial inequality, since the black population, in particular, is at greater risk of disparities in access to health services [23], which has resulted in a higher proportion of cases and deaths from Covid-19 in this population, a problem already evidenced in Brazil and the United States [24–27]. With regard to the obstetric population, a study carried out in 2020 with 12,556 pregnant and postpartum women with SARS due to Covid-19 in Brazil, showed that deaths from Covid-19 were twice as high in women who called themselves black, when compared to white women [28]. It is known that in Brazil, racial disparities in maternal mortality existed even before the pandemic, when the number of deaths among black, brown and indigenous women was disproportionately higher [29].

In view of this history and current evidence on racial disparities in health care in Brazil, and in view of the result of the present study, which identifies a greater chance of non-white women not being vaccinated, it is observed that racial disparities have also had an effect on vaccination against Covid-19. A study carried out in Maryland, USA, identified lower vaccination rates against Covid-19 when compared to regions with predominantly white populations [30].

It is known that the phenotypic characteristics of SARS-CoV-2 infection range from the complete absence of symptoms, mild and moderate clinical manifestations, to severe forms with possible pulmonary insufficiency and eventually death [31]. Incidentally, studies carried out in Brazil have identified a prevalence of pregnant and postpartum women with SARS who required ICU admission of approximately 20%, use of ventilatory support in 22%, and need for invasive ventilation ranging from 10% to 25% of cases [32, 33].

One of the objectives of vaccination is to prevent those infected from evolving into severe and critical cases of the disease [34]. Therefore, it is understood that the present findings on women who required ventilatory support and mechanical ventilation were more likely to be unvaccinated, demonstrating the possible positive impact of vaccination in the prevention of critical cases of the disease.

The only symptom that remained independently associated with the vaccination status of the study population was loss of taste, technically defined as ageusia, which in the present study was protective against non-vaccination. A study carried out with 417 patients with mild to moderate COVID-19 identified that 88% of the patients lost their sense of smell and taste during the virus infection [35]. It is hypothesized that the positive impact of vaccination on the severe course of the disease causes frequent symptoms to prevail among the vaccinated group in milder cases, such as ageusia.

This study has limitations, since it analyzes secondary data, where the lack of registration or the low quality of the data are common and cannot exclude the possibility of errors in the registration of the data. However, it is understood that the health information systems of the Brazilian Ministry of Health are of great value, as they have information that help in the planning of health policies and programs, contributing to the decision-making process. In addition, during the Covid-19 pandemic, SIVEP-Gripe has been the main source of data in the country.

Another limitation is that in this study, the group of vaccinated women consisted of all women who received at least one dose of the vaccine, regardless of the time interval between vaccination and the onset of signs and symptoms, which requires caution in interpretation of the results, since it is not an effectively immunized population.

In view of the findings, it is essential that health services advance in the vaccination of the obstetric population, especially adolescents, due to the low proportion identified. In addition, policies that address racial disparities are essential in order to contribute to improving the vaccination among black, brown and indigenous women. Vaccination against Covid-19 in the obstetric population already shows a positive impact on the evolution of severe cases, specifically, in cases of respiratory failure requiring ventilatory support and invasive ventilation, which reiterates the importance of vaccination against Covid-19.
